# BICM-ID Labeling-Based Recipient Identification in a Heterogeneous Network

**DOI:** 10.3390/s23073605

**Published:** 2023-03-30

**Authors:** Maciej Krasicki

**Affiliations:** Faculty of Computing and Telecommunications, Institute of Radiocommunications, Poznan University of Technology, Polanka 3, 61-131 Poznan, Poland; maciej.krasicki@put.poznan.pl; Tel.: +48-61-665-39-18

**Keywords:** coded modulation, iterative decoding, physical layer, turbo codes, wireless networks

## Abstract

The concept of labeling-based recipient identification (LABRID) for bit-interleaved coded modulation with iterative decoding (BICM-ID) is revisited. LABRID allows addressing a message recipient station in a wireless network by using an individual labeling map without compromising error performance. This eliminates the need to use any byte of the data frame to carry the recipient address explicitly. In addition, the destination of the frame can be determined in parallel with a BICM-ID decoding procedure in the receiver’s physical layer. Therefore, the MAC layer is not involved in processing the vast majority of frames transmitted in a network. Previously, it was shown that LABRID works fine if there are only LABRID-compatible stations within the network, and every receiver can reject frames destined for other receivers. This paper considers a scenario in which LABRID-compatible BICM-ID stations and legacy BICM stations coexist in the same network. A few experiments show that the LABRID receiver can reject an old-fashioned BICM frame by judging the convergence of the iterative decoding process. It also turns out that the legacy BICM receiver can identify and dismiss the LABRID-type frames thanks to the standard cyclic redundancy check (CRC) procedure.

## 1. Introduction

### 1.1. BICM-ID and LABRID Essentials

BICM-ID is an attractive solution for future wireless systems, transmitting over Rayleigh fading channels. Thanks to a feedback link between the channel decoder and the demapper, the BICM-ID receiver can substantially limit the number of errors within a frame as a result of the iterative decoding procedure, similar to the process known from turbo-code decoding. The success of iterative decoding depends on the labeling map (or simply, labeling) used to map binary blocks (i.e., parts of the codeword) onto the constellation points. Gray labeling, which is widely used in current wireless systems that employ original BICM, such as WLAN [1,2] and digital terrestrial television broadcasting [3,4], has been shown to be the worst choice from the perspective of iterative decoding goals. The optimal labeling maps for BICM-ID have been investigated in many papers, e.g., [5,6,7]. The most popular optimization target for labeling involves maximizing the asymptotic coding gain while maintaining the energy per bit $\left(E_{b}\right)$ to noise power density ratio $\left(N_{0}\right)$ at infinity. In such a case, the optimization criterion is to maximize the harmonic mean of the average distance between two constellation points, whose labels differ on exactly a one-bit position [5]. For the 16-QAM modulation, the asymptotically optimal labeling, found in [8], is called $M16^{a}$.

After careful observation of the properties of $M16^{a}$ labelings in [9], it was found that there are exactly 768 labelings possessing the features of $M16^{a}$, including both the asymptotic coding gain and the distance spectrum (which displays the distribution of the distances impacting the value of the asymptotic coding gain). This fact inspired the author of the current paper to investigate whether alternate use of different labelings of the same type could bring any benefit to a wireless system. The results, published in [10,11] for 16-QAM and 64-QAM modulation, respectively, showed that the receiver can filter out so-called "foreign frames", which are frames in which the symbols are mapped according to an asymptotically optimal labeling different from the one used at the receiver side. Therefore, it is possible to identify frame recipients using nothing but the receiver-specific labeling map. The process of discriminating foreign frames is based on the observation of the iterative decoding process convergence at the BICM-ID receiver. More specifically, it is enough to measure the absolute mean of the log-likelihood ratios (LLRs) outputted by the demapper (e.g., in the last iteration) and compare against a pre-defined threshold. This technique is called labeling-based recipient identification (LABRID).

One positive effect of LABRID is the possibility to resign from sending any physical address as a part of the data frame. However, taking into account the frame size (for BICM-ID, it should be large enough to effectively exploit the channel diversity [12]), the recipient identifier is usually just a small part of it (e.g., 48 bits for the conventional MAC “physical” address). In the case of a 1000-bit long data frame, it gives ca. 5% gain.

Telecommunication systems are usually designed according to the OSI [13] or TCP/IP [14] protocol stack pattern. In such cases, the destination address is buried in the MAC-layer frame. For a BICM-ID receiver it means that every physical data frame layer would have to be entirely decoded and transferred to the MAC layer, where its integrity would be checked (it is another decoding process), and then the recipient address would be read with the aim of eventually rejecting a frame if it appears to be foreign. For LABRID, the foreign frame is processed only in the physical layer. The BICM-ID channel decoder updates the extrinsic LLRs related to the codeword bits in every iteration, but the extrinsic LLRs associated with the data bits are calculated only once, i.e., in the last iteration; if the current frame is classified as foreign, it does not have to be decoded at all.

### 1.2. Current Research Problem Formulation

The results presented in [10,11] demonstrate the LABRID receiver’s ability to distinguish between foreign and desired frames in a homogeneous BICM-ID system with either 16-QAM or 64-QAM modulation, in which all stations are LABRID-compatible. This means that all receivers can run iterative decoding and use an appropriate labeling map. However, what happens if new LABRID-compatible stations are to co-exist with some legacy non-iterative BICM stations in a single network? Are the new receivers capable of rejecting the conventional BICM gray-labeled signals, transmitted between legacy stations? Another question of equal importance is if the signals mapped according to $M16^{a}$-equivalent labelings can be filtered out by any legacy BICM receiver. Note that such devices do not have any physical-layer mechanisms to assess frame destination. Instead, they must check the integrity of the MAC frame.

Finally, modern wireless systems incorporate the adaptive modulation strategy, which consists of setting the modulation order (and channel code) according to the current channel capacity. Therefore, receivers should be capable of rejecting signals of different modulation orders.

To study all of the problems outlined above, the author considers a heterogeneous simulation scenario with multiple transmitters and receivers (legacy or LABRID-compatible) and examines the efficiency of foreign frame rejection mechanisms.

The paper is organized as follows: Section 2 introduces the considered system model, including the description of the heterogeneous network and the structure of both LABRID-compatible BICM-ID and legacy BICM transceivers. In Section 3 the experiment results are reported. First, the bit-error rate (BER) vs. $E_{b}/N_{0}$ plots for both considered systems are examined to determine a reference $E_{b}/N_{0}$ range, in which the receiver’s ability to reject the foreign frames will be examined. The main part of the experiment consists of collecting a sufficient number of demapper output readings and analyzing their statistics. In Section 4, some conclusions are drawn.

## 2. System Model

### 2.1. Heterogeneous Network

**Figure 1:**
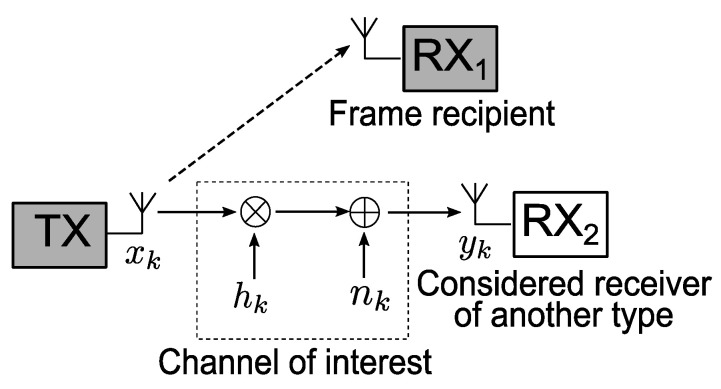
Considered channel model.

In the current paper, a heterogeneous network is considered, in which conventional BICM and LABRID-compatible BICM-ID stations coexist. The transmission is carried out between stations of the same type, but the signal may also reach one or more stations of the other type where it should be rejected. We study the receiver’s ability to reject the frame that is not destined for it. Therefore, as illustrated in Figure 1, we are interested in the channel between the transmitter of one type (TX) and the receiver of the other type (RX2) instead of the link between the stations of the same type (TX and RX1). The uncorrelated Rayleigh fading channel model is assumed; the sample received in the *k*th modulation period can be represented as

(1) $$y_{k}=h_{k}x_{k}+n_{k},$$

where $x_{k}$ is the signal element transmitted in the *k*th modulation period, $h_{k}$ (the channel gain) is a sample of the independent and identically distributed (i.i.d.) complex Gaussian random variable with zero mean and unit variance. In turn, $n_{k}$ is a sample of the additive white Gaussian noise, modeled as the i.i.d. complex Gaussian random variable with zero mean and $N_{0}$ variance.

### 2.2. LABRID-Compatible BICM-ID Station

**Figure 2:**
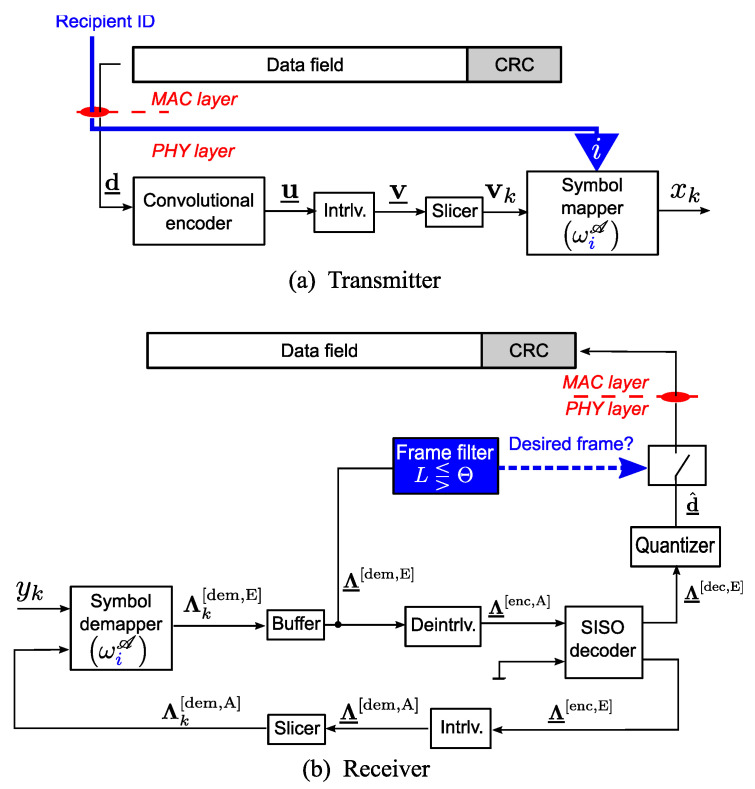
Block diagram of LABRID-compatible BICM-ID station.

A block diagram of the LABRID-compatible BICM-ID station is shown in Figure 2. The physical-layered 1000-bit long data frame, $\underline{d}$, which consists of the MAC-layer data field and 32 CRC bits and is dropped down from the MAC layer to the physical layer. It is passed to the input of a rate-1/2 ${\left[5\;\;7\right]}_{8}$ convolutional encoder, which is popular in BICM(-ID) applications. The encoder generates a codeword $\underline{u}$, which is interleaved by a random bit-wise interleaver of interleaving depth equal to the codeword size. The resultant interleaved codeword ($\underline{v}$) is sliced into *M*-bit long blocks, i.e., $v_{1},\ldots ,v_{K}$; each $v_{k}$ block is mapped onto a single constellation point by the symbol mapper. The labeling map, used for symbol mapping, depends on the recipient identifier, *i*, which is obtained from the MAC layer, according to the LABRID principles [10]. (For 16-QAM modulation, there are 768 asymptotically optimal labeling maps: $\omega_{1}^{A}\;\;,\cdots ,\omega_{768}^{A}$, which bring equally good BER vs. $E_{b}/N_{0}$ performance after iterative decoding.) The subsequent signal elements, $x_{1},\ldots ,x_{k},\ldots ,x_{K}$, are selected from the signal constellation in respective modulation periods $\left(x_{k}=\omega_{i}^{A}\;\left(v_{k}\right)\right)$, and are then transmitted over the channel.

In the receiver part of the LABRID-compatible station, a standard BICM-ID symbol demapper is used; it can process LLRs. It has two inputs: the first for the samples received from the channel $\left(y_{k}\right)$ and the second for $\Lambda_{k}^{\left[\mathrm{dem},A\right]}={\left[\Lambda_{k,m}^{\left[\mathrm{dem},A\right]}\right]}_{1\leq m\leq M}$, i.e., LLRs representing the *a priori* demapper ([dem,A] notation) beliefs on the values of individual bits of the *k*th block. The demapper calculates the so-called extrinsic LLRs for every *m*th bit of the *k*th block as

(2) $$\Lambda_{k,m}^{\left[\mathrm{dem},E\right]}=\log\frac{\sum_{\nu :\nu_{m}=0}\;\;\;\;\exp\;\left(\Gamma_{k,\nu }\;+\;\Lambda_{k}^{\left[\mathrm{dem},A\right]}\nu^{T}\;\right)}{\sum_{\nu :\nu_{m}=1}\;\;\;\;\exp\;\left(\Gamma_{k,\nu }\;+\;\Lambda_{k}^{\left[\mathrm{dem},A\right]}\nu^{T}\;\right)}-\Lambda_{k,m}^{\left[\mathrm{dem},A\right]},$$

where

(3) $$\Gamma_{k,\nu }=\log\frac{1}{\pi N_{0}}-\frac{1}{N_{0}}{\left|y_{k}-{\tilde{h}}_{k}\omega_{i}^{A}\;\left(\nu \right)\right|}^{2}$$

reflects the probability of receiving $y_{k}$, when a hypothetical ν block is transmitted, given the channel state $h_{k}$. More specifically, in (2), hypothetical *M*-bit long blocks ν with the *m*th bits equal to 0 and 1 are considered in the numerator and denominator, respectively. For such blocks, hypothetical signal elements are obtained as $\omega_{i}^{A}\;\left(\nu \right)$ and their distance to the received sample is calculated (including the estimated channel impact on the transmitted samples, represented by the channel estimate ${\tilde{h}}_{k}$).

The extrinsic demapper LLRs are buffered to obtain a single vector ${\underline{\Lambda }}^{\left[\mathrm{dem},E\right]}={\left[\Lambda_{k}^{\left[\mathrm{dem},E\right]}\right]}_{1\leq k\leq K}$, containing all LLRs related to the codeword. Afterward, this vector is deinterleaved (${\underline{\Lambda }}^{\left[\mathrm{enc},A\right]}$) and takes on the role of the soft-input soft-output (SISO) decoder’s *a priori* knowledge of the codeword (the encoded bits, as indicated by the LLRs’ superscripts). Due to the lack of space, the interested reader may refer to [15,16] for details about the SISO block routine. (Briefly, the SISO decoder operates according to the maximum *a posteriori* (MAP) principle and is accurate for both parallel and serial concatenated convolutional codes, as well as BICM(-ID). Depending on the system configuration, some inputs and/or outputs are not used. In the current scenario, there are no *a priori* beliefs on decoded data bits, so the respective input is tied to 0). The decoder updates its extrinsic LLRs related to the codeword, ${\underline{\Lambda }}^{\left[\mathrm{enc},E\right]}$, which are fed back through the interleaver to the demapper, and serve as the updated *a priori* knowledge, ${\underline{\Lambda }}^{\left[\mathrm{dem},A\right]}$, in the adjacent iteration.

In the last decoding pass, the SISO decoder calculates ${\underline{\Lambda }}^{\left[\mathrm{dec},E\right]}$, i.e., the extrinsic LLRs related to the data frame (or [dec]oded bits). As the MAC layer requires hard decisions on the data frame, denoted as $\underline{\hat{d}}$, the ${\underline{\Lambda }}^{\left[\mathrm{dec},E\right]}$ vector is passed through a quantizer that converts the input real-valued LLRs into a vector of unipolar symbols (or, simply, bits).

The key idea presented in the figure is to model a mechanism that blocks foreign frames from passing to the MAC layer. Similar to the first work on LABRID [10], this procedure is based on observing the convergence of the iterative process. Specifically, the mean of the absolute extrinsic demapper LLR per frame,

(4) $$\ell \equiv \langle \left|{\underline{\Lambda }}^{\left[\mathrm{dem},E\right]}\right|\rangle ,$$

is calculated in the last decoding pass; if it exceeds a pre-defined threshold, Θ, the frame is claimed to be a desired frame. Otherwise, it is rejected as a foreign frame, destined for another LABRID BICM-ID receiver.

To date, it has been shown that the frame filtering mechanism works properly if every received frame is LABRID-conformable (i.e., the symbols are mapped according to one of the asymptotically optimal labelings). In the current research, the LABRID receiver is confronted by a frame of gray-labeled symbols or a frame consisting of symbols mapped to another modulation order.

### 2.3. Conventional BICM Station

**Figure 3:**
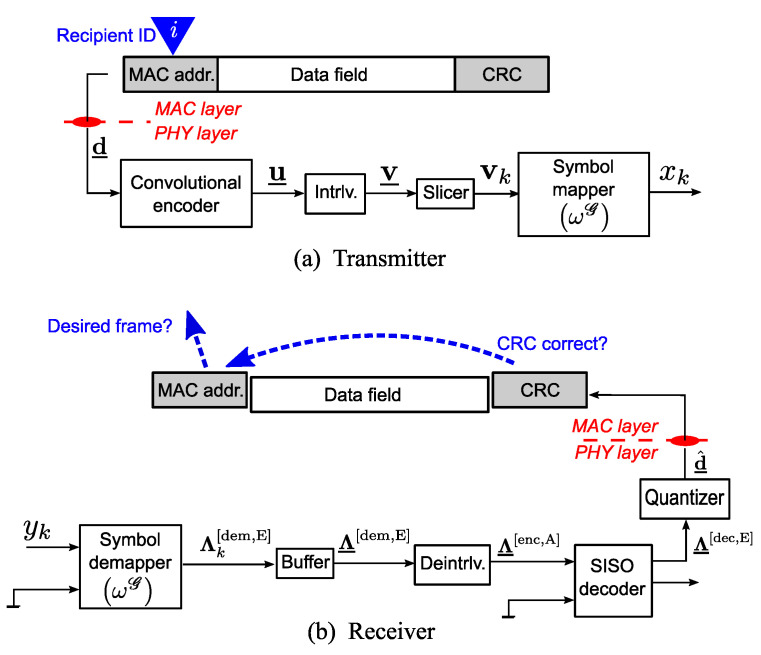
Conventional BICM station block diagram.

The structure of the legacy transceiver and receiver, considered in the current research, is shown in Figure 3.

It is assumed that there are hundreds of stations within the network, and the identifier of the current message recipient is *i* (it is unique within the network). However, in the conventional BICM system, the identifier is associated with the conservative 48-bit long MAC address, prepending the data field in the MAC-layer frame. The 32-bit CRC is then calculated, based on both the MAC address and the data field. The three components constitute a 1000-bit long physical-layer data frame, $\underline{d}$. In the physical layer, the signal processing is almost the same as in the LABRID-compatible station. The only difference is the labeling used when mapping the blocks onto the signal elements; in the current case, it is always the Gray labeling $\left(\omega^{G}\right)$, which is the optimal choice from the point of view of non-iterative BICM receivers in terms of their asymptotic coding gain [17].

With regard to the receiver, we use the same components as in the case of the BICM-ID. The demapper delivers soft bit-wise beliefs (in the LLR form) regarding the codeword. Formally, it calculates its extrinsic LLRs according to (2) with the assumption that all *a priori* LLRs are equal to 0. In practice, the formula can be simplified by removing the *a priori* messages. Obviously, Gray labeling is used instead of one of the asymptotically optimal labelings.

The SISO decoder runs just one time per data frame; it outputs the LLRs related to the data frame, denoted as ${\underline{\Lambda }}^{\left[\mathrm{dec},E\right]}$, which are passed through the quantizer. The vector of hard decisions, $\underline{\hat{d}}$, reaches the MAC layer. In the case of the conventional BICM receiver, every frame must be transferred to the MAC layer, where the CRC checksum is verified; finally, the destination of the frame can be derived from its MAC address. It is expected that if a foreign frame of the LABRID-type is received by the conventional BICM receiver, the CRC verification failure appears and, consequently, the frame is rejected without reading the destination MAC address.

## 3. Results

### 3.1. Reference BER vs. $E_{b}/N_{0}$ Plot

To study the LABRID-compatible receiver’s ability to reject the frames transmitted by the conventional BICM station, let us, first, specify the channel conditions under which 16-QAM LABRID BICM-ID is worth being used. For that goal, let both Tx and Rx stations be of the same (LABRID) type (as shown in Figure 2). The data frame of an affordable size (1000 bits) is still assumed. It is small enough to avoid huge delays and enormous memory requirements of the iterative decoding process. In total, as many as $10^{6}$ data frames are transmitted for statistical reliability reasons. The labeling map $\omega_{i}^{A}$ (the same for both the transmitter and receiver) is selected randomly from the set of all equivalent asymptotically optimal labelings. The ideal channel estimation is assumed, so ${\tilde{h}}_{k}=h_{k},\forall k$. All simulation parameters are grouped together in Table 1 for the reader’s convenience.

**Figure 4:**
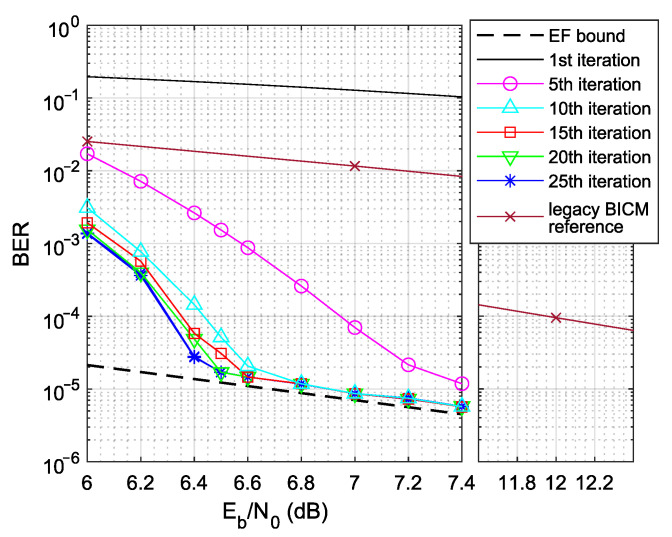
BER vs. $E_{b}/N_{0}$ for the 16-QAM LABRID BICM-ID system (data frame of 1000 bits).

Let us look at the BER vs. $E_{b}/N_{0}$ plot shown in Figure 4. The turbo-cliff, or waterfall (i.e., the region where the real BER curves start to decline rapidly to reach error-free feedback (EF) bound [5]), is quite flat, which is typical when relatively short data frames are assumed [18]. Our target BER is below a reasonable $10^{-4}$ level, which is reached at $E_{b}/N_{0}\approx 6.3\;\mathrm{dB}$ if all 25 iterations are executed, or for $E_{b}/N_{0}<6.4\;\mathrm{dB}$ after only 15 iterations. When compared with the first iteration line, the performance of the LABRID receiver is tremendous.

For reference, Figure 4 also contains the BER vs. $E_{b}/N_{0}$ plot for the legacy BICM system, the transmitter and receiver of which are shown in Figure 3. Note that the target $10^{-4}$ BER is reached at a much higher $E_{b}/N_{0}$ value (≈12dB).

### 3.2. Distribution of the Absolute Demapper Extrinsic LLR Mean

Now, let us assume the $E_{b}/N_{0}$ values that encompass the BICM-ID turbo cliff and study what happens if the LABRID-type BICM-ID receiver obtains a LABRID-incompatible signal. Since BER is not an accurate indicator of frame rejection efficiency, let us consider increments in the absolute demapper extrinsic LLR mean (4), which has been successfully used in the LABRID receiver to reject foreign frames in the homogeneous network scenario [10,11].

The first LABRID-incompatible signal to be studied is the 16-QAM signal generated by the legacy BICM station. The same data content and channel encoder are assumed as previously for the LABRID BICM-ID transmitter. The only difference is the Gray labeling used in the symbol mapper instead of any asymptotically optimal labeling map. The MAC address is replaced with random data (as in the previous setup) as they do not impact the LABRID mechanism.

**Figure 5:**
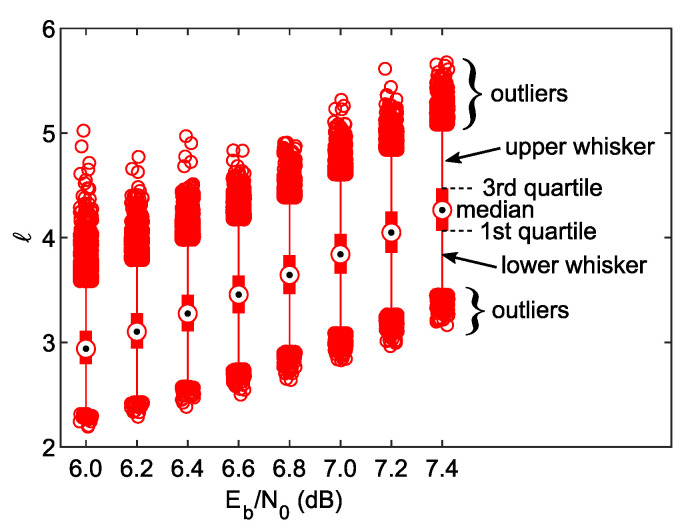
*ℓ* against $E_{b}/N_{0}$ statistics for the received foreign frames with the Gray-labeled signal in iteration 25.

Figure 5 shows the statistics of *ℓ* in the 25th iteration as a box plot [19]. This allows for easy identification of the median, the 1st and 3rd quartiles, as well as the range where almost all entries appear. The upper whisker ends at the value $q_{3}+1.5\cdot \left(q_{3}-q_{1}\right)$, and the lower whisker’s lower end is at $q_{1}-1.5\cdot \left(q_{3}-q_{1}\right)$, where $q_{1}$ and $q_{3}$ are the 1st and 3rd quartiles, respectively. For the Gaussian random variable distribution (in the considered case, the *ℓ* samples hold that feature under the central limit theorem) this gives coverage of ca. 99.3% of entries. Circles represent outliers, which exceed the range limited by the whiskers. To ensure statistical reliability, a large number of *ℓ* samples ($10^{6}$) have been collected for each $E_{b}/N_{0}$ point.

**Figure 6:**
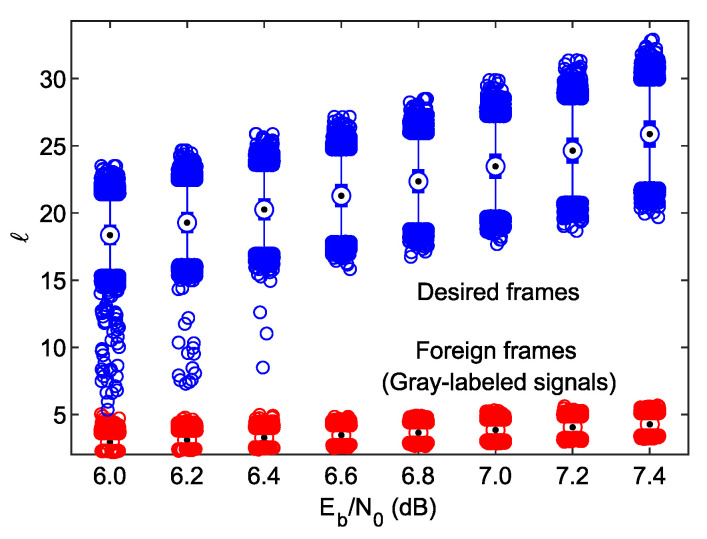
*ℓ* against $E_{b}/N_{0}$ statistics in iteration 25 for the received desired frames (blue) and foreign frames with Gray-labeled signal (red).

From Figure 5, it can be seen that the absolute extrinsic demapper LLR mean per frame grows with the $E_{b}/N_{0}$ ratio. It is expected because if the signal-to-noise ratio increases, the LLRs become more robust, as can be judged by (3). The crucial question is if one can specify any Θ threshold, such as ℓ<Θ for any foreign frame and ℓ>Θ for any desired frame. Let us sketch similar statistics for the case where the desired frame is received (it means that the labeling map used at the transmitter, $\omega_{i}^{A}\;$, suits that from the receiver). They are shown in Figure 6 and compared with those of the foreign frames, transmitted by the legacy BICM station. Now, in an appropriate scale, it is visible that the increment in the median of *ℓ* for the BICM frames, seen previously in Figure 5, is only marginal. From the current plot, one can conclude that at $E_{b}/N_{0}=6\;\mathrm{dB}$, it is hardly possible to discriminate foreign frames, as some outliers of both types overlap near ℓ=5. The author claims that it is caused by a “lazy” convergence of the iterative decoding process for a few LABRID-type frames, which might be a direct consequence of relatively short data frames and interleaver depth. However, judging by the BER vs. $E_{b}/N_{0}$ plot from Figure 4, the performance of the LABRID BICM-ID at $E_{b}/N_{0}=6\;\mathrm{dB}$ (above $10^{-3}$) lacks practicality. Meanwhile, for $E_{b}/N_{0}>6.4\;\mathrm{dB}$, there is a large space between the distributions of both systems’ *ℓ* values, so one can easily set up an appropriate threshold to distinguish between the foreign BICM and desired LABRID BICM-ID frames.

The above observations correspond with the conclusions in [10], where the LABRID receiver’s ability to reject the foreign LABRID-compatible frames was studied; the suggested optimal threshold ranges coincide with each other. When setting the threshold value, one must take into account a non-zero probability of the misdetection error, i.e., the event when a truly desired frame is considered a foreign frame. It limits the upper threshold bound to ca. 11.4, as stated in [10].

In conclusion, the LABRID receiver, ready to discriminate foreign frames, and destined to other LABRID-compatible receivers, can simultaneously reject any frame transmitted from the legacy BICM station with no extra condition or effort.

### 3.3. Early Decision Making

Subsequent iterations can bring some BER gain for the BICM-ID system, but it is desired to identify any foreign frame as soon as possible to save receiver power. Therefore, let us consider whether the decision to reject the foreign BICM frames can be made before the 25th iteration.

**Figure 7:**
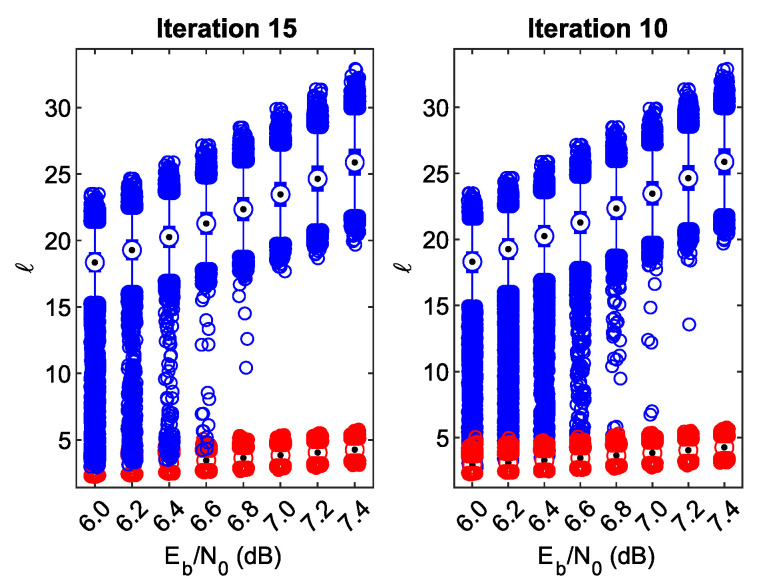
*ℓ* against $E_{b}/N_{0}$ statistics for the received foreign and desired frames in the 15th and 10th iterations (the meaning of individual data series as in Figure 6).

In Figure 7, the *l* distributions observed in the 15th and 10th iterations are shown in the respective subplots. From the figure, it is evident that making the decision in iteration 15 poses a problem if $E_{b}/N_{0}$ is below 7.0 dB, i.e., at $E_{b}/N_{0}=6.8\;\mathrm{dB}$ the minimal distance between the outliers of different types is much shorter than in Figure 6, and for any smaller $E_{b}/N_{0}$ they overlap each other, which leads to frame-filtering errors. In iteration 10, the situation is even worse—the outliers of different types are close to each other at $E_{b}/N_{0}=7\;\mathrm{dB}$, and they start to overlap at $E_{b}/N_{0}=6.8\;\mathrm{dB}$. The good news is that the *ℓ* distributions of the BICM foreign frames are almost identical, regardless of the considered iteration number, which proves that the iterative decoding process does not converge. In addition, judging by the whisker length, there is just a small number of desired BICM-ID frames exhibiting a low *ℓ* value. Based on these facts, an energy-preserving solution can be proposed as follows. In every iteration, we compare the calculated *ℓ* value against a relatively high threshold value (e.g., 8.0). If ℓ>Θ, the frame can be safely classified as desired and no further comparison is necessary. If not, the decision regarding the frame destination and type is postponed to the next iteration, and so on.

If the desired frame is recognized early, one can break the iterative process and decode the data frame immediately to save energy. The cost is a certain BER increase in the waterfall region—it can be seen in Figure 4.

### 3.4. LABRID Receiver Confronted by Random Signals

**Figure 8:**
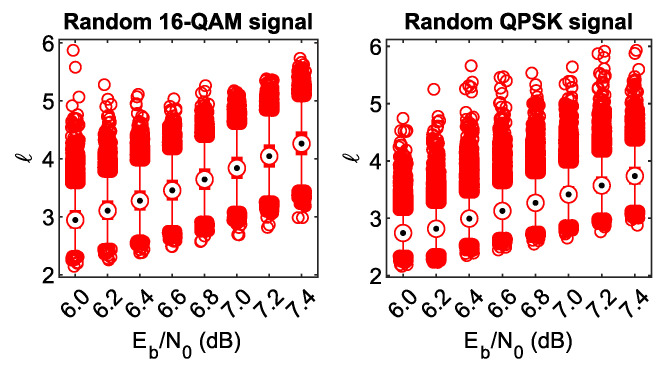
*ℓ* against $E_{b}/N_{0}$ statistics for the received random signals in iteration 25.

In this part, let us study the reception of a random 16-QAM or QPSK signal by the LABRID receiver. In this case, there is no channel encoder or interleaver at the transmitter side, and the signal elements are selected randomly from the signal constellation. Respective results are shown in Figure 8.

As expected, the iterative decoding process does not converge, which is reflected in poor *ℓ* statistics across the considered $E_{b}/N_{0}$ range. An important observation is that the distributions of *l* values at individual $E_{b}/N_{0}$ points for the random 16-QAM signal are very similar to their counterparts from Figure 5 (observe the median evolution from ca. 2.9 at $E_{b}/N_{0}=6.0\;\mathrm{dB}$ up to ca. 4.2 for $E_{b}/N_{0}=7.4\;\mathrm{dB}$). Thus, for the LABRID BICM-ID receiver, the Gray-labeled BICM signal, representing a valid codeword, means nothing more than the fully random 16-QAM signal and can be rejected with equal ease.

In the case of a received random QPSK signal, the situation is even better than for 16-QAM—note a generally slower median increment and a limited *ℓ* distribution width (the interquartile boxes are so short that they are hidden behind the median symbols).

### 3.5. On the Conventional BICM Receiver’s Ability to Reject LABRID Signal

Finally, we check whether the legacy BICM receiver can handle signals transmitted by the LABRID station. The former can determine the frame type by the result of the CRC verification: if the checksum is incorrect, it suggests that the frame is either of LABRID type or it is the desired legacy BICM frame but decoded incorrectly (in both cases, the frame is to be rejected).

In the current setup, we assume $E_{b}/N_{0}\in \left[10.0,\;12.0\right]$ with a 0.2 dB step (which is the region where BICM crosses the target $10^{-4}$ BER level, as seen in Figure 4), transmitting $10^{6}$ LABRID frames. As there is no plot to be displayed this time, let us just comment on the result: there was no frame with a valid checksum observed at any considered $E_{b}/N_{0}$ test point. Therefore, the conclusion is that the conventional BICM receiver can effectively dismiss LABRID frames.

## 4. Conclusions

In the paper, it is shown that the considered LABRID mechanism can be implemented in the heterogeneous network, in which BICM-ID and legacy BICM devices coexist. If the LABRID-compatible BICM-ID receiver obtains a frame transmitted by the legacy BICM station, it can detect the lack of iterative decoding convergence, which is reflected in a low value of the absolute extrinsic demapper LLR mean after several iterations. This value is not greater than what is observed for a random signal of the same or different modulation order. This suggests that the LABRID receiver could also function in systems with adaptive modulation order.

The LABRID-compatible signals do not disrupt the operation of the legacy BICM receivers; they can easily filter out inconsistent LABRID-type frames because such frames cannot pass CRC verification.

## Figures and Tables

**Table 1:** Simulation parameters’ specification.

Parameter	Value
Simulation method	Monte Carlo
Data frame size	1000 bits
Number of transmitted frames per $E_{b}/N_{0}$ point	106−
Channel model	uncorrelated Rayleigh fading
Target BER level	$10^{-4}$
Number of decoding iterations	15

## Data Availability

The data presented in this study are available on request from the corresponding author.
